# Mr. Chas Abbey

**Published:** 1882-02

**Authors:** 


*Death of Mr. Charles Abbey.*—Charles Abbey, whose name is inseparably associated with non-cohesive gold foil, is dead. If ever any man was a help to the dentist, Charles Abbey was he; and no dentist or manufacturer of dental goods need have a nobler monument than the old fashioned gold foil of Abbey & Sons' make for the man whose name it bears. Mr. Abbey was eighty-four years old, and was in the foil business for nearly seventy-five years, commanding the confidence of all who knew him.

